# Interoception and Empathy Impact Perspective Taking

**DOI:** 10.3389/fpsyg.2020.599429

**Published:** 2021-01-18

**Authors:** Lukas Heydrich, Francesco Walker, Larissa Blättler, Bruno Herbelin, Olaf Blanke, Jane Elizabeth Aspell

**Affiliations:** ^1^CORE Lab, Division of Psychosomatic Medicine, Department of Neurology, Inselspital, Bern University Hospital, University of Bern, Bern, Switzerland; ^2^Laboratory of Cognitive Neuroscience, Brain-Mind Institute, School of Life Sciences, Ecole Polytechnique Fédérale de Lausanne, Lausanne, Switzerland; ^3^Division of Psychosomatic Medicine, Department of Neurology, Inselspital, Bern University Hospital, University of Bern, Bern, Switzerland; ^4^Department of Psychology, University of Bern, Bern, Switzerland; ^5^Center for Neuroprosthetics, School of Life Sciences, Ecole Polytechnique Fédérale de Lausanne, Lausanne, Switzerland; ^6^Department of Neurology, Geneva University Hospitals, Geneva, Switzerland; ^7^School of Psychology and Sport Science, Anglia Ruskin University, Cambridge, United Kingdom

**Keywords:** empathy, interoception, perspective taking, bodily self-consciousness, illusion

## Abstract

Adopting the perspective of another person is an important aspect of social cognition and has been shown to depend on multisensory signals from one’s own body. Recent work suggests that interoceptive signals not only contribute to own-body perception and self-consciousness, but also to empathy. Here we investigated if social cognition – in particular adopting the perspective of another person – can be altered by a systematic manipulation of interoceptive cues and further, if this effect depends on empathic ability. The own-body transformation task (OBT) – wherein participants are instructed to imagine taking the perspective and position of a virtual body presented on a computer screen – offers an effective way to measure reaction time differences linked to the mental effort of taking an other’s perspective. Here, we adapted the OBT with the flashing of a silhouette surrounding the virtual body, either synchronously or asynchronously with the timing of participants’ heartbeats. We evaluated the impact of this cardio-visual synchrony on reaction times and accuracy rates in the OBT. Empathy was assessed with the empathy quotient (EQ) questionnaire. Based on previous work using the cardio-visual paradigm, we predicted that synchronous (vs. asynchronous) cardio-visual stimulation would increase self-identification with the virtual body and facilitate participants’ ability to adopt the virtual body’s perspective, thereby enhancing performance on the task, particularly in participants with higher empathy scores. We report that participants with high empathy showed significantly better performance during the OBT task during synchronous versus asynchronous cardio-visual stimulation. Moreover, we found a significant positive correlation between empathic ability and the synchrony effect (the difference in reaction times between the asynchronous and synchronous conditions). We conclude that synchronous cardio-visual stimulation between the participant’s body and a virtual body during an OBT task makes it easier to adopt the virtual body’s perspective, presumably based on multisensory integration processes. However, this effect depended on empathic ability, suggesting that empathy, interoception and social perspective taking are inherently linked.

## Introduction

Empathy is characterized as the ability to be sensitive to, to understand, and to experience the emotions of others (Ochsner et al., 2008; Singer et al., 2009; Reniers et al., 2011), requiring both affective/emotional and cognitive processes. Given that some authors have argued that emotion is an inherently embodied experience (Damasio, 2000), it has been suggested that individual differences in empathy might be linked to differences in the sensitivity to internal bodily signals (interoception). In line with this argument, there is evidence that individuals who are more sensitive to internal bodily signals tend to experience emotions more intensely and have a better understanding of their emotions (Critchley et al., 2004; Herbert et al., 2011). Empathy for pain has been linked to interoceptive accuracy (Grynberg and Pollatos, 2015) and a cortical index of interoception – the heartbeat evoked potential – correlates with self-reported empathic concern (Fukushima et al., 2011). Interoceptive awareness – measured via a questionnaire – was shown to correlate with both cognitive and affective aspects of empathy in a mixed group of individuals with and without autism (Mul et al., 2018). Interestingly, neuroimaging studies show that the insula, a primary interoceptive brain region, is also activated during the subjective awareness of feelings, including empathy (Ochsner et al., 2008; Craig, 2009; Singer et al., 2009; Zaki et al., 2012; Ernst et al., 2013), suggesting that learned associations between interoceptive signals and emotions observed in others may contribute to empathy (Bird and Viding, 2014; Quattrocki and Friston, 2014).

On the other hand, some authors have argued that empathy is strongly related to perspective taking ability because the ability to adopt the perspective of another person is an important aspect of social cognition (Adolphs, 2001), and perspective taking ability correlates with trait empathy (Mohr et al., 2010; Gronholm et al., 2012). One way to assess perspective-taking is the so-called own-body transformation task (OBT). In this task participants are asked to imagine adopting the perspective and position of a front- or back-facing virtual body presented on a screen, and to decide whether its marked hand would correspond to their own left or right hand (Parsons, 1987; Blanke et al., 2005). Longer reaction times are observed for front- as compared to back-facing figures, arguably due to the process of mental own body transformation required to imagine having perspective and position of the front- vs. back facing virtual body (Parsons, 1987; Blanke et al., 2005). This is further supported by the fact that the reaction times of mental transformation tasks also depend on the angle of rotation between the participant’s body and the imagined body or body part (Arzy et al., 2006a; Tadi et al., 2009; Heydrich et al., 2017).

There is evidence that spatial perspective taking involves a mental transformation of the observer’s own body, relying on motor, proprioceptive, and vestibular information, and it has been linked to multisensory brain regions (Blanke et al., 2005). During the OBT task, the brain regions that are selectively activated overlap with multisensory brain regions implicated in out-of-body experiences, e.g., the temporo–parietal junction (Blanke et al., 2005; Arzy et al., 2006b). Moreover, a patient who had out-of-body experiences was shown to behave differently in an OBT task (Overney et al., 2009).

We have previously shown that it is possible to modulate self-related processing and self-consciousness using a virtual body and visuo-tactile (Lenggenhager et al., 2007; Ionta et al., 2011) or visuo-interoceptive cues [cardio-visual stimulation (Aspell et al., 2013; Heydrich et al., 2018)]. In the latter case, presenting an illuminated outline around a virtual body flashing in synchrony with the participant’s heartbeat was found to increase illusory self-identification with the virtual body (the avatar’s body feels like one’s own body), shift self-location and alter somatosensory processing, as compared to an asynchronous control condition.

In the present study, we adapted the cardio-visual paradigm to an OBT task and aimed to investigate whether performance on a mental OBT task would be facilitated by online cardio-visual stimulation. To do this we visually projected heartbeat timing information onto the OBT virtual body (creating an illuminated outline around the virtual body), whose perspective participants were asked to take. We also tested whether this facilitatory effect would depend upon participants’ empathic ability measured via a validated empathy questionnaire (Baron-Cohen and Wheelwright, 2004).

## Methods

### Participants

A power calculation (*F* tests, ANOVA, repeated measures, within-between factors, assuming a power of 0.8; effect size of 0.3; significance level *α* = 0.05, four repetitions, correlation among repeated measures 0.5) using the software G^∗^Power (Faul et al., 2007) revealed that a sample size of at least 18 participants would be necessary. The estimation of a correlation of 0.5 was based on previous work using the OBT task where a correlation around 0.8 among repeated measures was found (Arzy et al., 2006b). A total of 20 healthy right-handed participants took part (nine females, mean age 26.7 ± 5.6 years). No participant had previous experience with the task or related experimental paradigms. All participants had normal or corrected to normal vision and had no history of neurological or psychiatric conditions. Participants gave written informed consent and were compensated for their participation. The study protocol was approved by the local Ethics Research Committee – La Commission d’éthique de la Recherche Clinique de la Faculté de Biologie et de Médecine – at the University of Lausanne, Switzerland and was performed in accordance with the ethical standards laid down in the Declaration of Helsinki.

### Materials and Procedure

#### Setup, Electrocardiogram (ECG), Signal Analysis

The present protocol adapted an experimental setup that has been used previously to study bodily self-consciousness and cardio-visual stimulation (Aspell et al., 2013; Heydrich et al., 2018). Raw data (ECG) were acquired with the BioSemi Active II^TM^ system (Biosemi, Netherlands) at a sampling rate of 2048 Hz. A custom signal processing software computed, at 60 Hz, the instantaneous derivative of the ECG signal (buffered data) to detect the high-amplitude signal change between the Q and R peaks of the ECG. Continuous adjustment of the algorithm to cumulatively averaged extrema allowed for an automatic adaptation to inter-participant differences and signal amplitude variations. Triggers were sent when the instantaneous signal change in the ascending Q to R phase of the ECG approached the averaged minimum to maximum amplitude difference (i.e., threshold of 90%). To guarantee maximum detection accuracy, ECG signals, and triggers were monitored (for largest amplitude and lowest noise) during the placement of the electrodes in order to find their optimal position on the chest, and the threshold was adjusted by the experimenter. A custom-made display software, created using Open Graphics Library, was programmed to superimpose a flashing outline onto the virtual body, that was characterized by a mean intensity of opacity (alpha) over the 800 × 600 pixels of 6%. In the synchronous condition, the silhouette surrounding the virtual body flashed for a duration of 100 ms and with a sinusoidal opacity of 0% starting at 0 ms to 100% at 50 ms and vice versa, aiming at a synchrony with the theoretical blood flow at the aortic root, and thus with the systolic heart contraction of the participant. The imprecision of the R-peak detection in the software is maximally 1 frame before or after the peak (33 ms). As the flash animation has a fixed duration of 100 ms, the “peak” of the flash therefore occurs on average at 73 ± 16 ms after the R peak [for details see Aspell et al. (2013)]. In the asynchronous conditions everything remained the same except that the virtual body/object was illuminated with a fixed delay of 400 ms with respect to the participant’s heartbeat (see Aspell et al., 2013; Heydrich et al., 2018).

#### OBT Task

**FIGURE 1:**
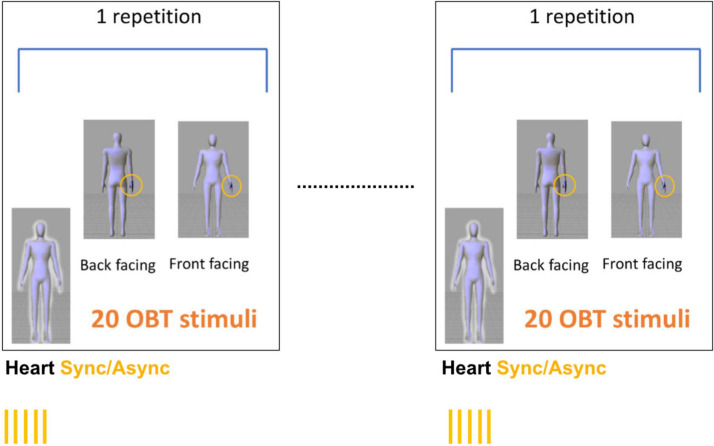
Stimulus presentation. Stimuli in the OBT task consisted of a schematic virtual body either facing toward (front facing) or away from the participant (back facing). The participants were asked to imagine themselves in the position and orientation of the virtual body displayed on the screen and to indicate which hand (right/left) was marked. Each experimental block consisted of five consecutive repetitions 20 OBT trials that were interleaved with periods of 35 s of seeing the virtual body with a flashing outline either synchronous (Heart Sync condition) or asynchronous (Heart Async condition) with respect to the heartbeat. This resulted in 100 OBT trials per experimental block. Because each experimental block was repeated twice per condition, a total of 400 OBT trials across conditions was recorded.

**FIGURE 2:**
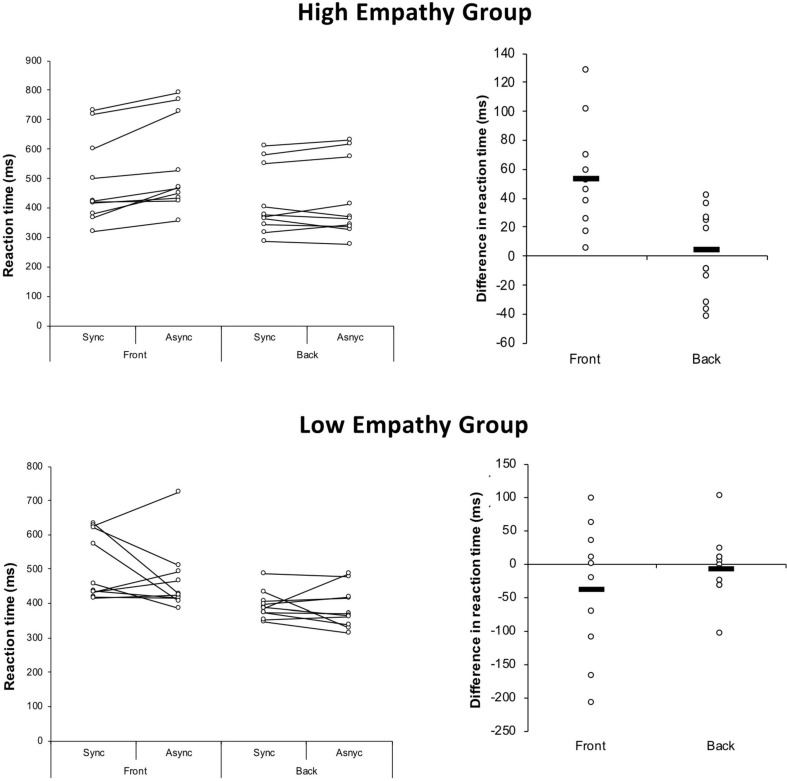
Results. Participants were split into low and high empathy scorers. Reaction times were significantly shorter if the virtual body was presented back-facing as compared to front facing. A significant interaction of synchrony and empathy group and a significant three-way interaction of synchrony, orientation, and empathy group was due to shorter reaction times in the condition in which the virtual body was flashing synchronously as compared to when the virtual body was flashing asynchronously in the high EQ group only.

Stimuli in the OBT task consisted of a schematic virtual body either facing toward (front facing condition) or away from the participant (back facing condition; Figure 1). Front and back figures had the same outline and were therefore scaled to the same proportions (dimensions: 2.8 × 6.1°). One of the virtual body’s hands was marked as if it were wearing a dark glove around the wrist, either on the right or left hand. Thus, there were a total of four figures: 2 (Front or Back) × 2 (right or left hand marked).

In the OBT task, the participants were asked to imagine themselves in the position and orientation of the virtual body displayed on the screen and to indicate which hand was marked. Stimuli were presented for 200 ms in the center of a computer screen. Participants sat at a distance of approximately 50 cm from the screen. Successive stimuli were only displayed after a response was given. The participants had to respond with their right hand as quickly and as accurately as possible – by pressing one of two buttons arranged vertically – to indicate whether the marked hand was the left or the right one.

#### Procedure

There were two different conditions, presented in a counterbalanced fashion between: (1) virtual body with flashing outline synchronous with the heartbeat (Heart Sync) and (2) virtual body with flashing outline asynchronous with the heartbeat (Heart Async).

For each condition we presented two blocks of 100 randomized (front/back) OBT trials. Within each block, five repetitions of 20 OBT trials were interleaved with 35 s periods of viewing the virtual body with an outline flashing synchronously (Heart Sync) or asynchronously with respect to the heartbeat (Heart Async, see Figure 1). This resulted in a total of 400 OBT trials across all conditions (i.e., 200 trials for Heart Sync and 200 trials for Heart Async). The duration of the experimental procedure was approximately 18–20 min per subject (without filling out the questionnaire).

#### Empathy

Empathy was assessed with the empathy quotient (EQ) questionnaire before the experimental procedure (Baron-Cohen and Wheelwright, 2004). The EQ comprises 60 questions: 40 items assessing empathy and 20 filler items. Each of the items were scored from 0 to 2 points (no, mild, strong empathic behavior), resulting in a maximum score of 80 and minimal score of 0 points.

#### Statistical Analysis

In order to assess the effect of synchronous cardio-visual stimulation and empathic ability on OBT performance, we conducted a two-tailed 3-way repeated-measures ANOVA with between-subject factor empathy (low/high) and within-factors orientation (front/back) and synchrony (synchronous/asynchronous). Participants were assigned to a low empathy group (median split, EQ Score < 42) or a high-empathy group. In order to follow up on the results of the ANOVA, a *t*-test (two-sided, paired) was used for *post hoc* testing and the significance (alpha) level used was *p* = 0.05 (corrected for multiple comparisons). The same statistic was applied to the accuracy data.

We also calculated a “synchrony effect size” by subtracting the reaction times for the synchronous condition from the asynchronous condition, e.g., yielding a positive value if the reaction time during the synchronous condition was shorter as compared to the asynchronous condition. We then used spearman’s correlation in order to assess the link between EQ and the synchrony effect size during front and back facing conditions separately.

## Results

### Own Body Transformation Task

We assessed the OBT effect and whether seeing a virtual body flashing synchronously or asynchronously with respect to the heartbeat as well as empathic ability had an influence on reaction times during the OBT task. Participants were split into low EQ (below median EQ score of 42, *n* = 10) and high EQ (above median EQ, *n* = 10) empathy scorers. The mean EQ Score was 41 points (SD ± 7.4). The median EQ Score was 42 points.

As predicted, statistical analysis revealed a significant effect of orientation [*N* = 20, *F*(1,18) = 56.271, *p* = 0.000, ηp2 = 0.758]. Thus, reaction times were significantly shorter if the virtual body was presented back-facing (mean reaction time = 406.2 ms, SD ± 105.2) as compared to front-facing (mean reaction time = 500.2 ms, SD ± 124.6, *p* < 0.001, see Figure 2). No main effect of synchrony [*N* = 20, *F*(1,18) = 0.084, *p* = 0.77, ηp2 = 0.004], empathy [*N* = 20, *F*(1,18) = 1.318, *p* = 0.25, ηp2 = 0.018] and no significant interaction between orientation and synchrony was found [*N* = 20, *F*(1,18) = 0.31, *p* = 0.58, ηp2 = 0.016].

However, we found a significant interaction of synchrony and empathy [*N* = 20, *F*(1,18) = 4.735, *p* = 0.043, ηp2 = 0.208] and a significant three-way interaction of synchrony, orientation and empathy [*N* = 20, *F*(1,18) = 9.308, *p* = 0.007, ηp2 = 0.341]. The latter was due to shorter reaction times in the condition in which a front facing virtual body was flashing synchronously (mean 487.78 ms, SD ± 146.41 ms) as compared to when the virtual body was flashing asynchronously (mean 541.83 ms, SD ± 159.04 ms, *t* = 4.50, df = 9, *p* = 0.007), in the high EQ group only. No significant difference was found for the back-facing condition in the high EQ group (synchronous condition: mean 420.20 ms, SD ± 100.18 ms; asynchronous condition: mean 424.66 ms, SD ± 103.64 ms, *t* = 0.49, df = 9, *p* = 0.6) nor for the low EQ group (front facing synchronous condition: mean 512.58 ms, SD ± 103.29 ms; asynchronous condition: mean 463.87 ms, SD ± 104.30 ms, *t* = −1.2, df = 9, *p* = 0.26; back facing synchronous condition: mean 395.17 ms, SD ± 43.98 ms; asynchronous condition: 376.54 ms, SD ± 52.00 ms, *t* = −0.4, df = 9, *p* = 0.69).

### Correlation

**FIGURE 3:**
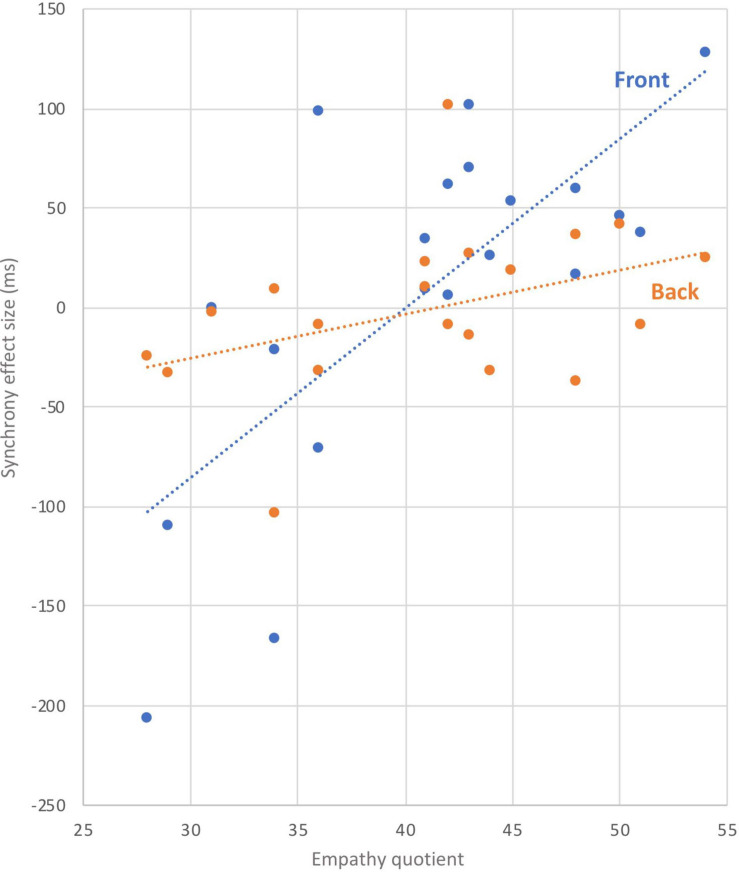
Correlation.

There was a significant correlation between EQ and “synchrony effect size” during the front-facing condition (rho = 0.69, dF = 18; *p* = 0.001, see Figure 3). No significant correlation was found between EQ and synchrony effect size during the back-facing condition (rho = 0.3, dF = 18, *p* > 0.05).

## Discussion

In the present study, we investigated whether performance on a mental OBT task would be modulated by online cardio-visual stimulation that has previously been shown to induce illusory changes in self-identification with a virtual body. We did this by visually projecting the participant’s heartbeat onto the virtual body that indicated the cue for the mental transformation. We further tested whether this potential modulatory effect would depend upon participants’ empathic ability. We found that for participants with high empathy ratings, reaction times during the OBT task were shorter for avatars in blocks where a flashing outline was synchronous with the participant’s heartbeat, as compared to blocks where a flashing outline was asynchronous with the heartbeat. This effect was only observed for the front facing virtual body (e.g., with higher OBT requirements) and was absent for the back facing virtual body. This was further corroborated by a significant correlation between EQ scores and the synchrony effect size for the front facing virtual body. Finally, OBT performance in low EQ participants was not modulated by the cardio-visual synchrony of the flashing outline.

Our findings support previous suggestions of a link between interoceptive processing and empathy (Fukushima et al., 2011; Herbert et al., 2011; Mul et al., 2018). Importantly, we were able to demonstrate that in participants with high empathic ability a marker of empathic ability –visuo-spatial perspective taking during the OBT task – can be systematically modulated by an online visualization of interoceptive activity –synchronous cardio-visual stimulation.

Why does synchronous cardio-visual stimulation result in an improved performance during the OBT task in participants with high empathic ability? Böffel and Müsseler (2018) have reported that perspective taking depends on perceived ownership over an avatar, while Ferri et al. (2011) have demonstrated that seeing one’s own hand as compared to someone’s else’s hand improves laterality judgments that involve a sensory-motor mental simulation.

We have previously shown that synchronous cardio-visual stimulation – i.e., presenting an illuminated outline flashing around a virtual body in synchrony with the participant’s heartbeat – results in increased self-identification with a virtual body, and alters somatosensory processing [cardio-visual full body illusion (Aspell et al., 2013; Heydrich et al., 2018)]. Although changes in bodily self-consciousness were not measured directly in the present study, based on previous findings (Aspell et al., 2013; Heydrich et al., 2018) we speculate that synchronous cardio-visual stimulation likely also enhanced self-identification with the virtual body and may have induced a drift in self-location toward the body during the OBT task, thereby improving visuo-spatial perspective taking ability required to imagine oneself in the position and orientation of the front facing body. Because this effect was absent for the back facing virtual body, where no visuo-spatial perspective shift is required, and since it was also absent for participants with lower empathic ability, synchrony-driven shifts in attention could be ruled out as an explanation. We also note there was no main effect of synchrony found by the analysis.

The effect of synchronous cardio-visual stimulation on visuo-spatial perspective taking was only present in participants with high empathic ability, while the correlation between EQ and the synchrony effect size for front facing avatars was observed across all participants. This suggests that the susceptibility to the effect of interoceptive cues on visuo-spatial perspective taking performance depends on empathic ability. This is in line with recent findings showing a link between empathy and the amplitude of the heartbeat-evoked potential (Fukushima et al., 2011) and the suggestion that intra-individual differences in empathy might be linked to differences in the sensitivity to internal bodily signals (Damasio, 2000). Our findings are also in keeping with evidence that people with higher empathic ability are more susceptible to a related multisensory (visuo-tactile) body illusion (Mul et al., 2019).

While visuo-spatial perspective taking and interoception have traditionally been considered to be based on distinct neural networks – i.e., perspective taking has been linked to the temporo–parietal junction (Blanke et al., 2005) and the representation of internal states to the insula (Craig, 2009) – here we show that the visual presentation of self-specific internal (cardiac) states alters performance of the OBT task in participants with high empathic ability. Based on this finding we speculate that in participants with high empathic ability the two systems are more tightly connected than in participants with low empathic ability. This would be in line with recent studies demonstrating that connectivity patterns depend on empathic ability (Esménio et al., 2019) and parieto-insular connectivity (Uddin et al., 2017). Stronger connectivity between parietal and insular regions would thus contribute to empathic ability by integrating both visuo-spatial perspective taking and processing of one’s internal states, including online interoceptive signals.

There are several limitations to the current study. Although a sample size calculation was performed in order to provide enough power for a repeated measures ANOVA, an even bigger sample size would have allowed us to perform a linear regression analysis, using EQ as a continuous predictor. Thus, a future study with a bigger sample is needed in order to confirm the robustness of these results. Also, the link between self-identification with the virtual avatar, synchronous cardio-visual stimulation and performance during the OBT task would need to be assessed directly using a questionnaire.

## Conclusion

Our finding that performance on a perspective taking task can be enhanced by synchronous cardio-visual stimulation and that this effect depends on empathic ability suggests that interoceptive processing, perspective taking and empathy are inherently inter-linked.

## Data Availability Statement

The raw data supporting the conclusions of this article will be made available by the authors, without undue reservation.

## Ethics Statement

The studies involving human participants were reviewed and approved by the La Commission d’éthique de la Recherche Clinique de la Faculté de Biologie et de Médecine – at the University of Lausanne, Switzerland. The participants provided their written informed consent to participate in this study.

## Author Contributions

LH and JA were responsible for the study design, data analysis, and writing of the manuscript. FW was responsible for data collection. LB helped with data analysis. BH helped with the study design and technical aspects of the setup. OB helped with the writing of the manuscript and supervision of the study. All authors contributed to the article and approved the submitted version.

## Conflict of Interest

The authors declare that the research was conducted in the absence of any commercial or financial relationships that could be construed as a potential conflict of interest.
